# Steroid metabolome analysis reveals prevalent glucocorticoid excess in primary aldosteronism

**DOI:** 10.1172/jci.insight.93136

**Published:** 2017-04-20

**Authors:** Wiebke Arlt, Katharina Lang, Alice J. Sitch, Anna S. Dietz, Yara Rhayem, Irina Bancos, Annette Feuchtinger, Vasileios Chortis, Lorna C. Gilligan, Philippe Ludwig, Anna Riester, Evelyn Asbach, Beverly A. Hughes, Donna M. O’Neil, Martin Bidlingmaier, Jeremy W. Tomlinson, Zaki K. Hassan-Smith, D. Aled Rees, Christian Adolf, Stefanie Hahner, Marcus Quinkler, Tanja Dekkers, Jaap Deinum, Michael Biehl, Brian G. Keevil, Cedric H.L. Shackleton, Jonathan J. Deeks, Axel K. Walch, Felix Beuschlein, Martin Reincke

**Affiliations:** 1 Institute of Metabolism and Systems Research, University of Birmingham, Birmingham, United Kingdom.; 2 Centre for Endocrinology, Diabetes and Metabolism, Birmingham Health Partners, Birmingham, United Kingdom.; 3 Institute for Applied Health Research, University of Birmingham, Birmingham, United Kingdom.; 4 Medizinische Klinik und Poliklinik IV, Ludwig-Maximilians-Universität München, Munich, Germany.; 5 Division of Endocrinology, Metabolism and Nutrition, Department of Internal Medicine, Mayo Clinic, Rochester, Minnesota, USA.; 6 Research Unit Analytical Pathology, Helmholtz Zentrum Munich, Oberschleißheim, Germany.; 7 Oxford Centre for Diabetes, Endocrinology and Metabolism, University of Oxford, Oxford, United Kingdom.; 8 Neurosciences and Mental Health Research Institute, School of Medicine, Cardiff University, Cardiff, United Kingdom.; 9 Department of Medicine I, Endocrine and Diabetes Unit, University Hospital Würzburg, Würzburg, Germany.; 10 Endocrinology in Charlottenburg, Berlin, Germany.; 11 Department of Internal Medicine, Radboud University Medical Centre, Nijmegen, Netherlands.; 12 Johann Bernoulli Institute for Mathematics and Computer Science, University of Groningen, Groningen, Netherlands.; 13 Department of Clinical Biochemistry, University Hospital South Manchester, Manchester, United Kingdom.; 14 University of California at San Francisco Benioff Children’s Hospital, Oakland, California, USA.

## Abstract

**BACKGROUND.** Adrenal aldosterone excess is the most common cause of secondary hypertension and is associated with increased cardiovascular morbidity. However, adverse metabolic risk in primary aldosteronism extends beyond hypertension, with increased rates of insulin resistance, type 2 diabetes, and osteoporosis, which cannot be easily explained by aldosterone excess.

**METHODS.** We performed mass spectrometry–based analysis of a 24-hour urine steroid metabolome in 174 newly diagnosed patients with primary aldosteronism (103 unilateral adenomas, 71 bilateral adrenal hyperplasias) in comparison to 162 healthy controls, 56 patients with endocrine inactive adrenal adenoma, 104 patients with mild subclinical, and 47 with clinically overt adrenal cortisol excess. We also analyzed the expression of cortisol-producing CYP11B1 and aldosterone-producing CYP11B2 enzymes in adenoma tissue from 57 patients with aldosterone-producing adenoma, employing immunohistochemistry with digital image analysis.

**RESULTS.** Primary aldosteronism patients had significantly increased cortisol and total glucocorticoid metabolite excretion (all *P* < 0.001), only exceeded by glucocorticoid output in patients with clinically overt adrenal Cushing syndrome. Several surrogate parameters of metabolic risk correlated significantly with glucocorticoid but not mineralocorticoid output. Intratumoral CYP11B1 expression was significantly associated with the corresponding in vivo glucocorticoid excretion. Unilateral adrenalectomy resolved both mineralocorticoid and glucocorticoid excess. Postoperative evidence of adrenal insufficiency was found in 13 (29%) of 45 consecutively tested patients.

**CONCLUSION.** Our data indicate that glucocorticoid cosecretion is frequently found in primary aldosteronism and contributes to associated metabolic risk. Mineralocorticoid receptor antagonist therapy alone may not be sufficient to counteract adverse metabolic risk in medically treated patients with primary aldosteronism.

**FUNDING.** Medical Research Council UK, Wellcome Trust, European Commission.

## Abstract 2

Glucocorticoid excretion in primary aldosteronism patients is increased and correlates with both clinical metabolic risk parameters and intra-tumoral expression of the cortisol-producing enzyme CYP11B1.

## Introduction

Primary aldosteronism is the most common form of secondary hypertension, affecting 3%–5% of the general hypertensive population and 8%–10% of patients referred to specialist hypertension services (1–4). The main causes of primary aldosteronism are bilateral adrenal hyperplasia and unilateral aldosterone-producing adrenal adenoma (1), whereas inherited forms of primary aldosteronism, such as familial hyperaldosteronism type 1 and 3, account for less than 1% of cases. In the majority of aldosterone-producing adenomas, somatic driver mutations in the *KCNJ5*, *ATP1A1*, *ATP2B3*, *CACNA1D*, and *CTNNB1* genes have been identified (5–9). Differentiating unilateral aldosterone-producing adenoma from bilateral adrenal hyperplasia as the cause of primary aldosteronism provides the basis for therapeutic stratification. Adenomas are generally surgically removed, while patients with bilateral hyperplasia receive long-term treatment with mineralocorticoid receptor antagonists.

Hypertension is a strong predictor of cardiovascular morbidity and mortality (10). Patients with primary aldosteronism have an even higher cardiovascular risk than patients with essential hypertension (11–15). However, in addition, a number of reports have described an increased prevalence of insulin resistance, type 2 diabetes mellitus, osteoporosis, as well as clinically relevant depression and anxiety in patients with primary aldosteronism (16–23). These conditions would be more logically attributable to glucocorticoid rather than mineralocorticoid excess. Current diagnostic work-up in primary aldosteronism, however, does not include assessment for hypercortisolism (1).

Here, we employed mass spectrometry to explore the steroid metabolome in a large cohort of primary aldosteronism patients who underwent detailed metabolic phenotyping, in addition to subtype specification and genotyping. We compared the results of 24-hour urine steroid metabolite excretion, reflective of net steroid production, to those in healthy controls and patients with adrenal adenomas including subclinical and overt adrenal Cushing’s syndrome.

## Results

### Demographics and clinical metabolic phenotype.

**Figure 1:**
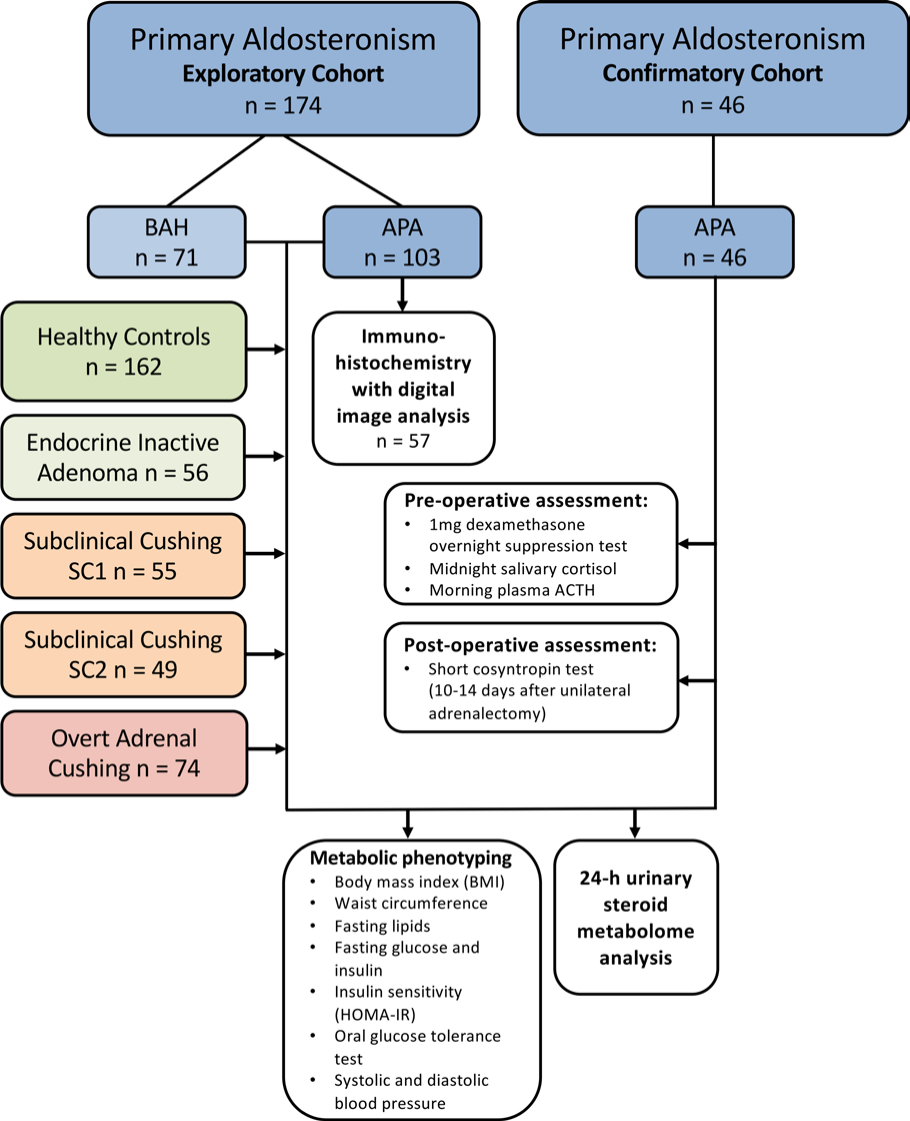
CONSORT diagram visualizing the included patient and comparator groups and the performed diagnostic work-up. After analysis of the results from the exploratory cohort, 46 patients were prospectively recruited with consecutive enrollment into the confirmatory cohort that underwent additional pre-and postoperative assessment of cortisol production. APA, unilateral aldosterone-producing adenoma; ACTH, adrenocorticotropic hormone; BAH, bilateral adrenal hyperplasia; HOMA-IR, homeostasis model assessment of insulin resistance.

We evaluated an exploratory cohort of 220 consecutively recruited patients with primary aldosteronism: an exploratory cohort comprising 103 patients with unilateral aldosterone-producing adenoma, 71 patients with bilateral adrenal hyperplasia, and a confirmatory cohort of 46 patients with unilateral adenoma. For comparison, we included 162 healthy control subjects and 3 additional patient groups: 56 patients with endocrine-inactive adrenal adenoma; 104 patients with subclinical, mild adrenal cortisol excess; and 47 patients with clinically overt adrenal Cushing syndrome. The clinical characteristics of all study subjects are summarized in Table 1. Following the analysis of the exploratory cohort results, we prospectively recruited a confirmatory cohort of 46 patients with primary aldosteronism; Figure 1 provides a flowchart showing the patient and comparator groups and the diagnostic assessments they underwent.

### Steroid metabolome analysis in primary aldosteronism.

**Figure 2:**
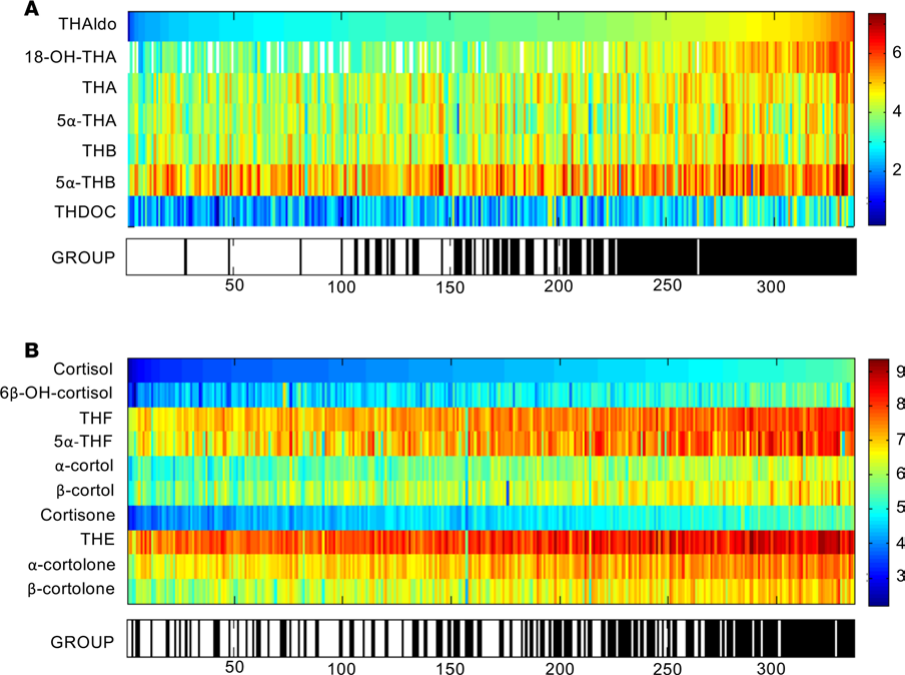
Heatmap visualizations of steroid metabolome profiling results in 174 primary aldosteronism patients. Steroid metabolite excretion in 24-hour urine was measured by gas chromatography-mass spectrometry in selected-ion-monitoring mode. Heatmap visualizations depict log-transformed steroid metabolite excretion values in the primary aldosteronism patients (group: black) in comparison to 162 healthy controls (group: white). (**A**) Mineralocorticoid and mineralocorticoid precursor metabolites ordered according to increasing amounts of tetrahydroaldosterone (THAldo) excretion. (**B**) Glucocorticoid metabolites in order of increasing amounts of cortisol excretion. Scale and color code were chosen separately for each panel according to the respective range of observed values. THA, tetrahydro-11-dehydrocorticosterone; THB, tetrahydrocorticosterone; THDOC, tetrahydro-11-deoxycorticosterone; THF, tetrahydrocortisol; THE, tetrahydrocortisone.

A heatmap visualization sorted in the order of increasing excretion of the major aldosterone metabolite tetrahydroaldosterone (Figure 2A) demonstrated, as expected, a clustering of the primary aldosteronism cases to the right of the heatmap, consistent with increased aldosterone production. Surprisingly, an equivalent heatmap visualizing glucocorticoid output, sorted in the order of increasing cortisol excretion, also demonstrated a clear clustering of primary aldosteronism patients among those with the highest glucocorticoid output (Figure 2B).

As expected, 24-hour tetrahydroaldosterone excretion was significantly increased in primary aldosteronism patients (healthy controls, median 30 [interquartile range 22–44] μg/24 h; primary aldosteronism, 99 [interquartile range 60–159] μg/24 h; *P* < 0.001) (Supplemental Information [SI] Appendix and Supplemental Figure 1A; supplemental material available online with this article; https://doi.org/10.1172/jci.insight.93136DS1). However, quantitative comparison of steroid excretion between primary aldosteronism patients and healthy controls also revealed 24-hour total glucocorticoid output as significantly increased in primary aldosteronism (11,306 [interquartile range 7,999–14,907] μg/24 h vs. 8,262 [6,337–11,070] μg/24 h in healthy controls; *P* < 0.001), with similar findings for cortisol excretion (SI Appendix and Supplemental Figure 1, B and C).

Both primary aldosteronism subtypes, unilateral aldosterone-producing adrenal adenoma and bilateral adrenal hyperplasia, had significantly higher glucocorticoid output than controls, with no significant difference between the 2 disease groups (SI Appendix and Supplemental Figure 2). Similarly, comparison of glucocorticoid excretion in patients with adenomas harboring somatic *KCNJ5* mutations (*n* = 31) did not differ from those with non-*KCNJ5* mutations (*n* = 23) and wild type (*n* = 34), with significantly increased glucocorticoid excretion in all 3 (data not shown).

### Glucocorticoid output in primary aldosteronism and adverse metabolic risk phenotype.

Correlation analyses adjusted for sex and age revealed 24-hour total glucocorticoid output as significantly correlated with body mass index and markers of insulin resistance (fasting insulin, insulin after oral glucose challenge; homeostasis model assessment of insulin resistance [HOMA-IR]) in both control subjects and primary aldosteronism (all *P* < 0.01). However, in primary aldosteronism patients, total glucocorticoid output also significantly correlated with additional parameters of adverse metabolic risk (waist circumference [*P* < 0.001], high-density lipoprotein [*P* < 0.001], and diastolic blood pressure [*P* < 0.01]; Supplemental Table 1). By contrast, tetrahydroaldosterone excretion only correlated with systolic blood pressure, albeit above our stringent level of significance (*P* ≤ 0.01), with a *P* value of 0.02 (Supplemental Table 1).

### Steroid output in primary aldosteronism in comparison to Cushing’s patients.

**Figure 3:**
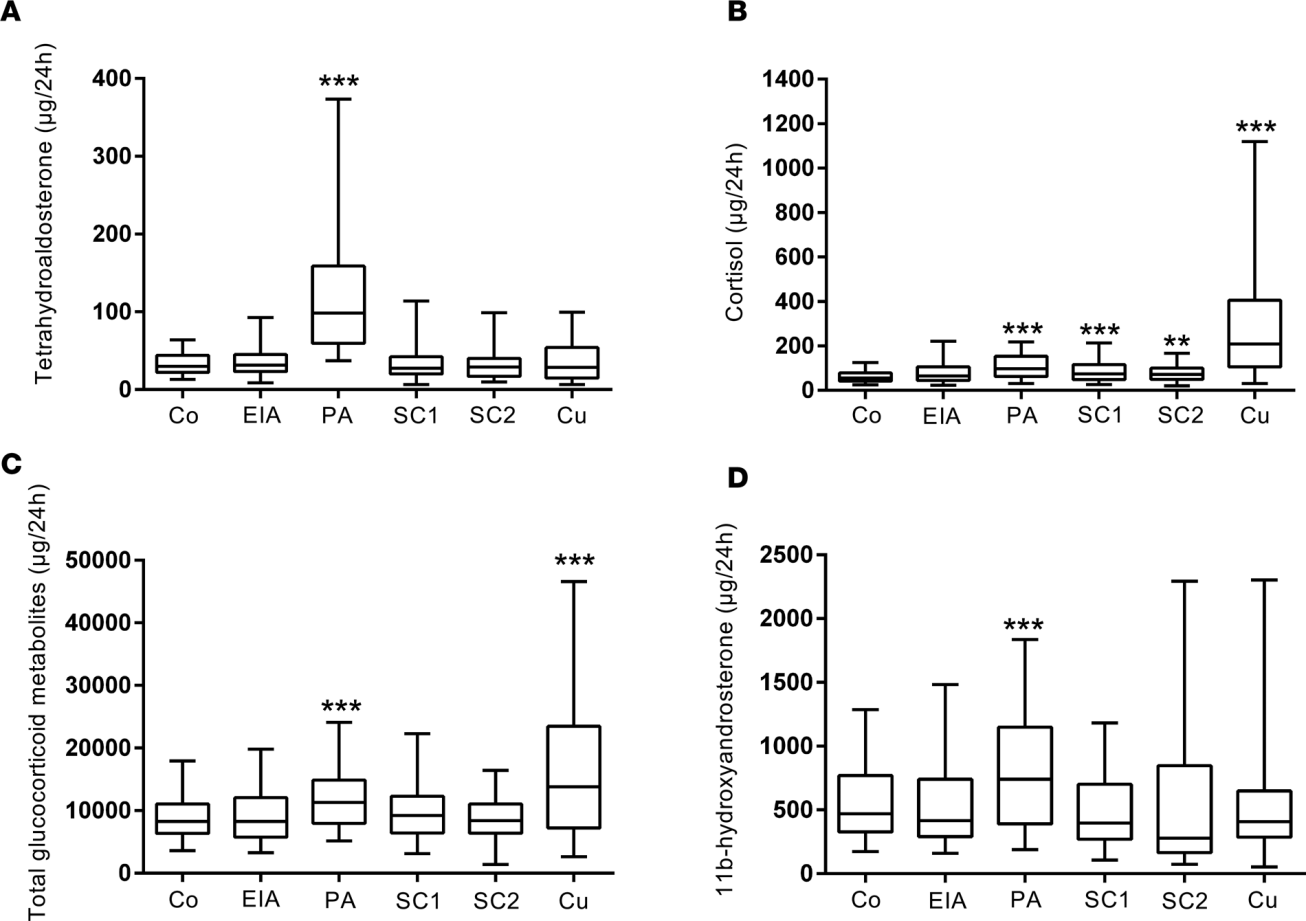
Steroid metabolite excretion in primary aldosteronism in comparison to healthy controls and patients with endocrine-inactive and cortisol-producing adrenal adenomas. The panels show the 24-hour urinary excretion of tetrahydroaldosterone (**A**), cortisol (**B**), total glucocorticoid metabolites (**C**), and the major adrenal androgen metabolite 11β-hydroxyandrosterone (**D**) in primary aldosteronism patients (PA; *n* = 174) in comparison to healthy controls (Co; *n* = 162), patients with endocrine-inactive adrenal adenoma (EIA; *n* = 56), patients with subclinical Cushing’s (differentiated into 2 groups: SC1 (*n* = 55), morning cortisol after 1 mg dexamethasone overnight > 50 and < 138 nmol/l; SC2 (*n* = 49), morning cortisol >138 nmol/l), and overt adrenal Cushing’s syndrome patients (Cu; *n* = 47). Boxes represent median and interquartile range, whiskers represent 5th and 95th centiles. **P < 0.01 versus controls, ***P < 0.001 versus controls. Comparisons between groups were made with linear regression models to adjust for age and sex in comparisons between all 6 groups.

To put the extent of glucocorticoid excess into context, we compared glucocorticoid output in the primary aldosteronism patients to that in patients with adrenocortical adenomas that were either endocrine inactive or fulfilled the diagnostic criteria for subclinical and clinically overt Cushing’s syndrome (Figure 3, SI Appendix, and Supplemental Table 2). This revealed significantly increased cortisol output in primary aldosteronism that was at least as high as in patients with subclinical Cushing’s syndrome, a group with documented adverse cardiovascular and metabolic consequences due to mild cortisol excess (24–26). Furthermore, total glucocorticoid excretion was significantly higher in primary aldosteronism (median 11,306 [interquartile range 7,999–14,907] μg/24 h) than in controls (8,262 [6,337–11,070] μg/24 h), while subclinical Cushing’s patients did not significantly differ from controls (SI Appendix and Supplemental Table 2). The percentage difference in total glucocorticoid excretion relative to healthy controls was 25.0% (95% CI 14.4, 35.6) in primary aldosteronism and only 3.4% (95% CI –21.4, 28.1) in patients with subclinical Cushing’s syndrome (SI Appendix and Supplemental Table 3).

A detailed pathway analysis of the steroid metabolome in the 4 different disease groups revealed distinct differences (SI Appendix and Supplemental Figure 3). The primary aldosteronism group showed significantly increased excretion of all metabolites of the mineralocorticoid and glucocorticoid synthesis pathways. Their androgen production largely resembled those in controls but showed an increased excretion of the major adrenal androgen metabolite 11β-hydroxyandrosterone (SI Appendix and Supplemental Figure 3A). This was found in primary aldosteronism, but none of the other groups (Figure 3D). By contrast, the overt Cushing’s group (SI Appendix and Supplemental Figure 3B) showed decreased androgen output in the presence of increased glucocorticoid and mineralocorticoid precursor metabolites. The subclinical Cushing’s groups (SI Appendix and Supplemental Figure 3, C and D) showed a similar pattern to overt Cushing’s, albeit attenuated with only 2 of 9 glucocorticoid metabolites significantly increased. Patients with primary aldosteronism and overt Cushing’s patients showed significant increases in all 9 glucocorticoid metabolites (SI Appendix and Supplemental Figure 3, A and B).

### Steroid output in primary aldosteronism due to unilateral adenoma before and after adrenalectomy.

**Figure 4:**
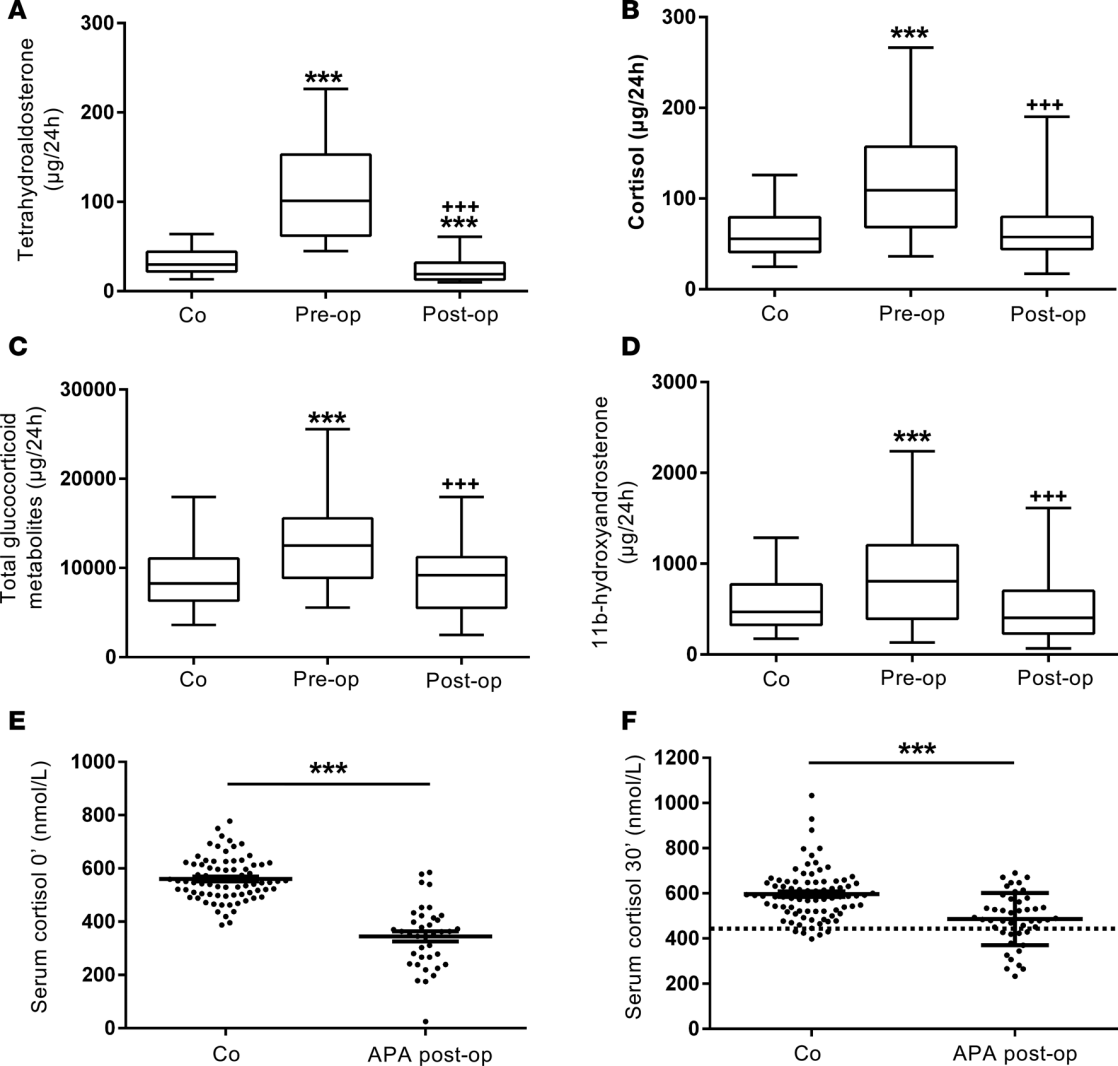
Steroid excretion in 46 patients with primary aldosteronism due to aldosterone-producing adenoma (APA) before and after unilateral adrenalectomy in comparison to healthy controls (*n* = 162). The panels show 24-hour urinary excretion of tetrahydroaldosterone (**A**), cortisol (**B**), total glucocorticoid metabolites (**C**), and 11β-hydroxyandrosterone (**D**). ***P < 0.001 versus controls (Co); ^+++^*P* < 0.001 versus preoperative. Boxes represent median and interquartile range, whiskers represent 5th and 95th centiles. Panels **E** and **F** show individual values and mean ± SEM for serum cortisol at baseline and 30 minutes after cosyntropin stimulation in 46 primary aldosteronism patients tested 2 weeks postoperatively, in comparison to 82 healthy controls. The dotted line represents the diagnostic cut-off for adrenal insufficiency (serum cortisol 30 minutes after 250 μg cosyntropin < 15.6 μg/dl [<430 nmol/l], equivalent to the 5th centile of the cortisol response in the 82 healthy controls). ***P < 0.001 versus controls. Comparisons of steroid excretion before and after unilateral adrenalectomy were made using 2-sided Wilcoxon’s signed-rank test. Linear models were also fitted comparing preoperative and postoperative log-transformed steroid-metabolite measures to controls, adjusting for age and sex.

Comparing preoperative to postoperative steroid excretion in a cohort of 46 patients with primary aldosteronism due to unilateral aldosterone-producing adenoma, we found that following surgical removal of the adenoma, not only tetrahydroaldosterone but also total glucocorticoid output significantly decreased (Figure 4, A–C). Similarly, excretion of 11β-hydroxyandrosterone, the metabolite of the major adrenal androgen precursor 11β-hydroxyandrostenedione, significantly decreased after surgery (Figure 4D, SI Appendix, and Supplemental Table 4). Heatmap visualization comparing pre- and postoperative urine steroid metabolomes revealed patient clusters with increased mineralocorticoid and glucocorticoid production prior to surgery (SI Appendix and Supplemental Figure 4, A and B), but also demonstrated distinct, albeit smaller clusters of patients with preoperatively increased 11β-hydroxyandrosterone (SI Appendix and Supplemental Figure 4C). This suggested that the source of glucocorticoid and adrenal androgen excess in these patients was indeed the surgically removed adrenocortical adenoma.

### Intratumoral steroidogenic enzyme expression and correlation with in vivo steroid output.

**Figure 5:**
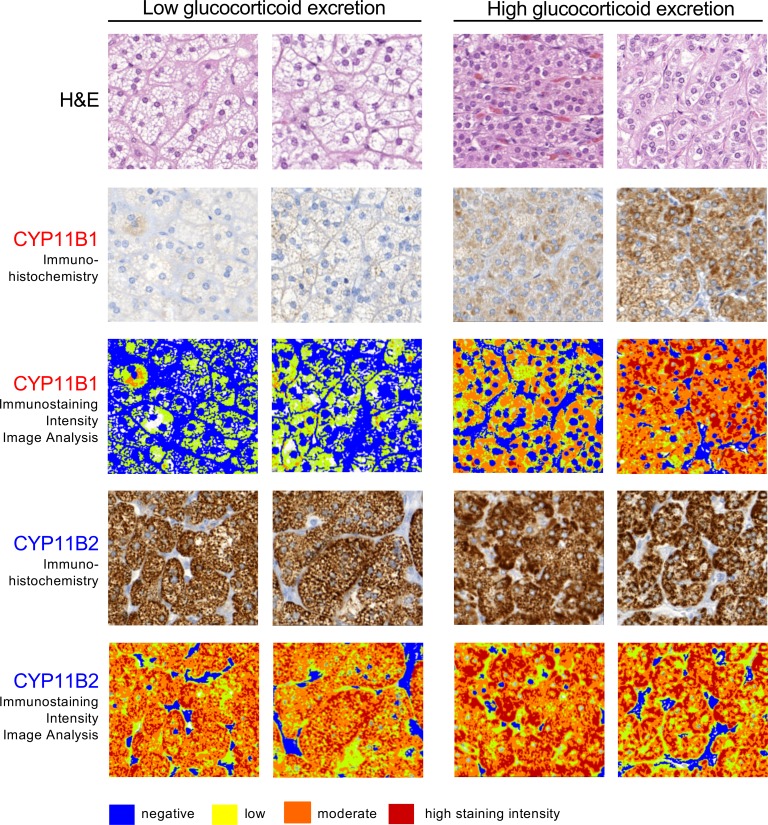
Immunohistochemistry with digital image analysis for steroidogenic enzyme expression in aldosterone-producing adrenal adenoma tissue in relation to in vivo 24-hour glucocorticoid excretion. A tissue microarray with adenoma tissue from 57 patients was studied by immunohistochemistry for expression of CYP11B1, required for cortisol and 11β-hydroxyandrostenedione synthesis, and CYP11B2, the enzyme responsible for aldosterone synthesis, followed by digital image analysis for quantification of staining intensity. Representative immunohistochemistry examples from 2 patients with in vivo glucocorticoid excretion within the lowest and highest quartiles, respectively, are shown (total original magnification in all panels, ×20).

We next carried out immunohistochemistry of a tissue microarray composed of surgical material from 57 of our aldosterone-producing adrenal adenoma patients and quantified intratumoral steroidogenic enzyme expression by digital image analysis. We analyzed the expression of CYP11B1, which is required for the synthesis of cortisol and 11β-hydroxyandrostenedione, and CYP11B2, which catalyzes aldosterone synthesis. Results showed that CYP11B1 immunostaining was associated with the excretion of glucocorticoids (*P* < 0.05) and the major 11β-hydroxyandrostenedione metabolite 11β-hydroxyandrosterone (*P* < 0.01), but not with tetrahydroaldosterone. Vice versa, CYP11B2 was associated with tetrahydroaldosterone (*P* < 0.05), but not with glucocorticoid or 11β-hydroxyandrosterone excretion (Table 2). Of note, tumor morphology consistently differed when comparing adenoma tissues in relation to glucocorticoid output, with a zona fasciculate–type pattern in tissues from patients with the lowest glucocorticoid excretion, and a pattern resembling zona glomerulosa architecture in tissue from patients with the highest glucocorticoid excretion (examples shown in Figure 5).

### Glucocorticoid excess in primary aldosteronism and postoperative cortisol reserve.

After the analysis of our results suggested that glucocorticoid excess represents a prevalent and highly relevant feature in primary aldosteronism, we prospectively assessed the pre- and postoperative adrenal function in a confirmatory cohort of 46 consecutively recruited patients with unilateral aldosterone-producing adenoma. Interestingly, despite significantly increased urinary glucocorticoid output, very few patients showed results indicative of glucocorticoid excess, using either an overnight dexamethasone suppression test or late-night salivary cortisol. However, preoperatively, plasma corticotropin (andrenocorticotropic hormone, ACTH) was low normal, with a median of 14 [interquartile range 9–19] pg/ml, which increased significantly (*P* = 0.0012) to 22 [interquartile range 16–39] pg/ml 2 weeks postoperatively (reference range 4–50 pg/ml).

Clinically relevant glucocorticoid excess due to an adrenal tumor is typically associated with postoperative suppression of the hypothalamic-pituitary-adrenal axis and hence suppression of function of the contralateral adrenal gland. This results in the need for hydrocortisone replacement in patients with subclinical and overt adrenal Cushing’s syndrome after curative adrenalectomy (27). Therefore, 46 consecutively recruited patients underwent a cosyntropin stimulation test to assess cortisol reserve 10–14 days after removal of the aldosterone-producing adrenocortical adenoma. Consistent with the evidence of glucocorticoid excess in the steroid metabolome analysis, 13 of 45 (29%) patients failed to appropriately respond to cosyntropin stimulation (serum cortisol 30 minutes after 250 μg ACTH_1–24_ < 15.6 μg/dl [<430 nmol/l]), with both baseline and stimulated serum cortisol significantly lower in primary aldosteronism than in 82 healthy controls (*P* < 0.01) (Figure 4, E and F).

## Discussion

A number of previous case reports or small case series (28–35) have reported excess glucocorticoid secretion in primary aldosteronism patients, a phenomenon thought to represent a rarity. However, the current study provides evidence for the concomitant presence of glucocorticoid excess in a large proportion of patients with primary aldosteronism, an observation we found to be independent of primary aldosteronism subtype or tumor tissue genotypes. Of note, we found that glucocorticoid output in primary aldosteronism was at least similar to that in subclinical Cushing’s syndrome, a cohort widely reported to suffer from increased cardiovascular risk (24–26). Intriguingly, we found that glucocorticoid excess significantly correlated with several parameters indicative of adverse metabolic risk in primary aldosteronism, including waist circumference, high-density lipoprotein, and diastolic blood pressure, in addition to body mass index and insulin resistance that were influenced by obesity in both controls and primary aldosteronism patients. By contrast, there was no significant association of mineralocorticoid output with metabolic risk parameters. This suggests that the previously unrecognized glucocorticoid excess in primary aldosteronism is a major determinant of metabolic risk. This potentially novel biochemical characteristic of primary aldosteronism could provide an explanation for comorbidities that are observed but difficult to explain in the context of hyperaldosteronism, such as insulin resistance (16–20), osteoporotic fractures (21, 36), and depression (22, 23). Patients with primary aldosteronism who do not undergo surgery currently receive medical treatment with mineralocorticoid receptor antagonists. Our findings indicate that it should be investigated whether such patients might need additional, glucocorticoid-opposing treatment to mitigate persistent cardiovascular risk. That this is clinically highly relevant is further supported by a recent cohort study in 2,533 patients with primary aldosteronism, which found a particularly increased osteoporotic fracture risk in women with primary aldosteronism who were not treated surgically but medically with mineralocorticoid receptor antagonists (37).

In autonomous adrenal cortisol excess, i.e., adrenal Cushing’s syndrome, adrenal androgens are usually downregulated due to reduced hypothalamic-pituitary feedback to the adrenal glands in response to the glucocorticoid excess. However, steroid metabolome analysis in our large primary aldosteronism cohort revealed not only glucocorticoid excess, but normal or increased androgen output. Intriguingly, steroid pathway analysis revealed evidence for additional autonomous production of 11β-hydroxyandrostenedione, which is a major product of adrenal steroidogenesis (38) and a direct precursor to potent androgens (39, 40). Steroid pathway analysis results were strongly corroborated by the results of quantitative immunohistochemistry in aldosterone-producing adenoma tissue, demonstrating a significant association of intratumoral expression of the CYP11B1 enzyme, required for the synthesis of both glucocorticoids and 11β-hydroxyandrostenedione, with in vivo excretion of the corresponding steroid metabolites. Intriguingly, cosecretion of glucocorticoids could also potentially explain the recent finding that adrenal vein sampling performs less well than previously assumed in differentiating unilateral from bilateral adrenal causes of primary aldosteronism (41), as this test relies on the assumption of an increase in the aldosterone over cortisol ratio in unilateral disease, which may be diluted in adenomas with cosecretion.

Glucocorticoid excess in mild, subclinical adrenal Cushing’s syndrome is associated with overnight dexamethasone test results that are regularly abnormal, while 24-hour urine glucocorticoid excretion is often normal. By contrast, we found that glucocorticoid excess in primary aldosteronism regularly presented with increased 24-hour urine glucocorticoid output preoperatively and one-third of patients showed evidence of compromised cortisol reserve postoperatively. However, only a few patients failed the overnight dexamethasone test and at present the reason for this is unclear. Patients with primary pigmented nodular adrenocortical disease, another adrenal cause of glucocorticoid excess, do not suppress after dexamethasone either, but have been shown to paradoxically increase glucocorticoid production in response to dexamethasone (42).

The high rate of patients failing cosyntropin testing following surgical removal of their aldosterone-producing adrenal adenoma, 29% of consecutively recruited subjects, was confirmed in direct comparison to healthy controls measured by the same mass spectrometry–based assay. Adrenal insufficiency has not been appreciated as likely to occur postoperatively in primary aldosteronism, which in light of our findings now will have to be reconsidered. In our current experience, postoperative adrenal insufficiency is transient, mostly recovering after 3 to 6 months. However, during this period patients are exposed to the potentially fatal risk of adrenal crisis as they are not able to appropriately increase their cortisol production in case of major illness or surgery. A recent study (43) was the first to assess the impact of unilateral adrenalectomy itself on cortisol reserve, documenting normal responses to cosyntropin testing in 30 live kidney donors tested on postoperative days 1 and 28. One would have anticipated these results, given that the physiological response to adrenalectomy will be a counter-regulatory upregulation of ACTH, stimulating cortisol production in the remaining adrenal gland. Therefore, those recent findings further corroborate that the high rate of failed responses to cosyntropin in our primary aldosteronism patients cannot be explained by mere loss of adrenal tissue volume, but will be a consequence of preoperative cortisol excess, resulting in reduced responsiveness of the contralateral adrenal and hence reduced cosyntropin-stimulated cortisol output.

### Conclusions.

Our findings demonstrate that glucocorticoid excess is a highly prevalent feature in primary aldosteronism and closely linked to adverse metabolic risk; we also show evidence of autonomous adrenal androgen production in primary aldosteronism. Steroid metabolome analysis also demonstrated increased mineralocorticoid output in the Cushing comparator cohort, suggesting that the traditional division into Cushing’s and Conn’s is not as clear cut as previously assumed. Steroid metabolomics is a highly sensitive diagnostic and biomarker tool in adrenal disease (44, 45), including primary aldosteronism (46, 47); our study now demonstrates the value of steroid metabolome analysis as a discovery tool for the dissection of disease mechanisms. Further studies will have to assess the clinical impact of the discovery of “Connshing” syndrome, including prospective randomized studies to clarify whether primary aldosteronism patients should receive additional glucocorticoid-opposing treatment when treated medically with mineralocorticoid receptor antagonists to efficiently counteract adverse metabolic risk.

## Methods

### Patients and controls.

Patients and controls were recruited at 5 centers participating in the European Network for the Study of Adrenal Tumors. All participants provided written informed consent, and the study was approved by the ethics committee at each participating institution.

At the time of diagnosis, all primary aldosteronism patients underwent systematic metabolic and cardiovascular phenotyping. Metabolic phenotyping was also carried out in healthy controls and the other comparator cohorts. Primary aldosteronism patients were compared to 5 comparator cohorts (Table 1): healthy controls (*n* = 162); patients with endocrine-inactive adrenal adenoma (*n* = 56); patients with adrenal adenomas associated with mild, subclinical Cushing’s syndrome (*n* = 104); and patients with overt clinical Cushing’s syndrome (*n* = 47) due to a cortisol-producing adrenal adenoma. In the latter 3 groups, imaging, histopathology, and follow-up investigations had confirmed the benign nature of the adrenal lesion.

The diagnosis of corticotropin-independent adrenal Cushing’s syndrome was based on a combination of clinical and biochemical features of hypercortisolism. Patients were classified as suffering from overt Cushing’s syndrome if they had at least 3 abnormal biochemical test results indicative of Cushing’s, in addition to insufficiently suppressed serum cortisol levels (>5 μg/dl [>138 nmol/l]) after overnight administration of 1 mg of dexamethasone. Biochemical tests considered indicative of Cushing’s were increased urinary excretion of free cortisol, increased late-night salivary cortisol levels, and suppressed plasma corticotropin levels (<10 pg/ml [<2.2 pmol/l]).

Overt Cushing’s syndrome was also diagnosed if the patients had at least 2 biochemical results indicative of Cushing’s in the presence of typical catabolic signs of hypercortisolism (i.e., muscle weakness, skin fragility, osteoporosis). Patients without typical catabolic signs, nonsuppressible serum cortisol levels, and up to 2 other abnormal biochemical tests were diagnosed as subclinical Cushing’s. For the purposes of our analysis, we divided the subclinical Cushing’s patients into 2 groups, in line with recently published consensus diagnostic criteria (48): possible autonomous cortisol excess, SC1, and autonomous cortisol excess, SC2, defined by serum cortisol after overnight administration of 1 mg of dexamethasone (SC1: > 1.8 and ≤ 5.0 μg/dl [> 51 and ≤ 138 nmol/l]); SC2: > 5.0 μg/dl [>138 nmol/l]). Patients were considered to have endocrine-inactive adrenal lesions if they had normal biochemical test results and no catabolic signs.

To independently confirm the findings arising from the exploratory primary aldosteronism cohort, we prospectively recruited 46 consecutive patients diagnosed with lateralized aldosteronism undergoing unilateral adrenalectomy. In addition to 24-hour urine steroid profiling, they underwent preoperative assessment for glucocorticoid excess including dexamethasone (1 mg) overnight suppression test, late-night salivary cortisol, and morning plasma ACTH; all patients underwent a postoperative cosyntropin test (serum cortisol at baseline and 30 minutes after intravenous injection of 250 μg ACTH_1–24_, Synacthen) 10–14 days after unilateral adrenalectomy.

### Genetic analysis.

In 88 patients who underwent adrenalectomy for aldosterone-producing adenoma, DNA was extracted from tumor tissue as described previously (49). DNA was subjected to targeted sequencing of genes previously identified to harbor somatic mutations in aldosterone-producing adenomas (*KCNJ5*, *ATP1A1*, *ATP2B3*, *CACNA1D*, and *CTNNB1*), as described previously (49). Thereby, tumor tissues from 54 patients were found to harbor somatic mutations in the following genes: *KCNJ5* (*n* = 31), *ATP1A1* (*n* = 8), *ATP2B3* (*n* = 4), *CACNA1D* (*n* = 6), *CTNNB1* (*n* = 1), PRKACA (*n* = 2) (50), and *CACNA1D*+*CTNNB1* (*n* = 2). Tumor tissue in the residual 34 patients did not contain any of these 5 somatic candidate mutations.

### Clinical and biochemical metabolic phenotyping.

Baseline anthropometric assessment in the patients with primary aldosteronism included height, body mass index (kg/m^2^), and standardized blood pressure measurement using an automatic sphygmomanometer validated for clinical use (according to list on www.dableducational.org). For blood pressure, the mean of 3 measurements separated by 5 minutes was determined and used for calculations.

Blood samples were drawn for fasting plasma glucose and insulin, hemoglobin A_1c_ (HbA_1c_), total cholesterol, high-density lipoprotein, and triglycerides. Most participants (*n* = 107) also underwent a 75-g oral glucose tolerance test with blood sampling at 30-minute intervals for 2 hours. Subjects were categorized as having normal glucose tolerance on the basis of 2-hour glucose values (normal glucose tolerance < 140 mg/dl [<7.8 mmol/l], impaired glucose tolerance ≥ 140 mg/dl [≥7.8 mmol/l]). HOMA-IR was calculated using the following formula: [fasting glucose (mmol/l) × fasting insulin (mIU/l)]/22.5.

### Urine steroid metabolome analysis.

All patients collected a 24-hour urine at the time of diagnosis of primary aldosteronism for analysis by quantitative gas chromatography-mass spectrometry (GC-MS) in selected-ion-monitoring (SIM) analysis mode, as described previously (45). For urinary steroid metabolite excretion analysis by quantitative GC-MS in SIM analysis mode, urinary steroids were enzymatically released from conjugation, with subsequent recovery of the hydrolyzed steroids by C18 solid-phase extraction (SPE) using Sep-Pak columns (Waters), followed by chemical derivatization prior to GC-MS-SIM analysis. A subcohort of 46 patients also collected 24-hour urine 6 to 12 months after unilateral adrenalectomy.

Our method allows for identification and quantification of 32 different steroid metabolites derived from mineralocorticoids, glucocorticoids, androgens, and their precursors; details are summarized in Supplemental Table 2. 3α,5β-Tetrahydroaldosterone (THAldo) represents the major mineralocorticoid metabolite. Total glucocorticoid production was assessed as the sum of quantified metabolites of cortisol and cortisone, comprising tetrahydrocortisol (THF), 5α-tetrahydrocortisol (5α-THF), tetrahydrocortisone, α- and β-cortol, α- and β-cortolone, 6β-hydroxycortisol, and urinary cortisol and cortisone.

### Hormonal assessments.

We used validated and established liquid chromatography-tandem MS assays to measure serum cortisol (51), serum dexamethasone (52), and salivary cortisol (53). Cosyntropin test results were compared to those obtained in 82 healthy controls measured with the same assay; the diagnostic cut-off for diagnosis of adrenal insufficiency was defined as the 5th centile of the healthy control cohort and determined as 15.6 μg/dl (430 nmol/l). Plasma ACTH was measured in EDTA plasma samples using a commercially available, automated chemiluminescence immunoassay (Liaison, Diasorin).

Serum dexamethasone concentrations greater than 2.2 μg/dl (>5.6 nmol/l) were considered indicative of a valid overnight dexamethasone suppression test. Salivary cortisol assay reference ranges were 0.2–1.7 μg/dl (5–46 nmol/l) in the morning and less than 0.1 μg/dl (<2.6 nmol/l) in the evening. Plasma ACTH was measured in EDTA plasma samples using the Liaison chemiluminescence immunoassay. The assay employs 2 monoclonal antibodies directed against the N- and C-terminal end. Cross-reactivity with ACTH precursors and fragments is negligible. In our hands, the limit of quantification is 4 pg/ml with a reportable range from 4 to 1,500 pg/ml. Intra-assay coefficients of variability (CVs) ranged from 1.2% to 15.0% at concentrations of 355 and 5.8 pg/ml, while interassay CVs were 7.3% at 195 pg/ml and 12.7% at 14 pg/ml.

### Tissue microarrays, immunohistochemistry, and digital image analysis.

Tissue microarrays were constructed by sampling 3 tumor tissue cores (1.0 mm in diameter) as previously described (54). Paraffin-embedded tissue blocks from 57 of the aldosterone-producing adenomas were utilized, which were selected on the basis of representative H&E–stained tissue sections of each case by the same pathologist (AW).

The technical construction of the tissue microarrays was performed on a manual tissue arrayer (AlphaMetrix Biotech). A semi-automated rotation microtome (Microm HM 355S, Thermo Fisher Scientific) was used to cut 3-μm tissue microarray sections that were transferred to glass slides for immunohistochemistry and H&E staining.

Immunohistochemical staining was performed under standardized conditions on a Discovery XT automated stainer (Roche Diagnostics/Ventana Medical Systems) employing monoclonal antibodies directed against human CYP11B1 and CYP11B2 (55), provided by Celso Gomez-Sanchez, University of Mississippi Medical Center, Jackson, Mississippi, USA. The slides were incubated with primary antibodies (rat monoclonal anti-hCYP11B1 [1:20], clone 80-2-2 or mouse monoclonal anti-hCYP11B2 [1:100], clone 41-17B in Dako REAL antibody dilution, catalog S2022), and detected by the Discovery DAB Map Kit (Roche Diagnostics/Ventana Medical Systems), including incubation with respective secondary antibodies (anti-rat, Vector, catalog BA 4001 [1:300]; anti-mouse, ready-to-use universal secondary antibody, Roche Diagnostics/Ventana Medical Systems, catalog 760-4205).

Following immunohistochemistry, stained tissue microarray slides were scanned at ×20 objective magnification using a digital Mirax Desk slide scanner (Zeiss Microimaging) prior to import into the image analysis software Definiens Developer XD2 (Definiens AG), as previously described (56). Tumor areas were annotated manually and a rule set was defined to detect and quantify staining intensities in the annotated tumor area, with operators blinded with regard to corresponding in vivo steroid output.

### Statistics.

Summary statistics, comprising mean (standard deviation) and median [interquartile range] (dependent on the presence of outliers), and heatmap visualization, were used to describe the clinical characteristics and the steroid metabolome of the patients included in this study. Initial analysis was undertaken using Mann-Whitney *U* tests comparing patients with primary aldosteronism and healthy controls; patients with unilateral adrenal adenoma and bilateral adrenal hyperplasia; and patients with primary aldosteronism, endocrine inactive adrenal adenoma, subclinical adrenal Cushing’s (SC1 and SC2) and overt adrenal Cushing’s, with healthy controls.

Further analysis used linear regression models to adjust for age and sex in comparisons between all 6 groups, and additionally adjust for body mass index in comparisons of primary aldosteronism and healthy controls, using log-transformed outcome data to reduce the impact of high outliers. Estimates from the regression model are presented with 95% confidence intervals and interpreted as approximate percentage differences (57). For the primary aldosteronism patients and the control subjects, separately, the association between patient characteristics and both tetrahydroaldosterone and total glucocorticoid metabolites adjusted for age, sex, and body mass index differences was assessed by fitting linear regression models. Log-transformed outcome data were used to reduce the impact of high outliers, and estimates interpreted as approximate percentage changes per unit change in the characteristic (57).

Comparisons of steroid excretion before and after unilateral adrenalectomy were made using Wilcoxon’s signed-rank test. Linear models were also fitted comparing preoperative and postoperative log transformed steroid-metabolite measures to controls, adjusting for age and sex. For the 57 patients with aldosterone-producing adenoma immunohistochemistry data, analyses were performed to assess the association between total glucocorticoid metabolites, 11β-hydoxyandrosterone and tetrahydroaldosterone, and the level from image analysis. Again, linear regression with log-transformed outcome data was used and estimates can be interpreted as approximate percentage changes.

All tests were 2-sided and a stringent *P* value (*P* ≤ 0.01) was considered as indicative of statistical significance given the exploratory nature and number of comparisons made in the analysis. The software packages R, Stata version 13, and MATLAB were used for analysis.

### Study approval.

Patients and controls were recruited at 5 centers participating in the European Network for the Study of Adrenal Tumors. All participants provided written informed consent, and the study procedures were approved by the ethics committee at each participating institution.

## Author contributions

WA, FB, and MR designed research. WA, KL, AR, EA, ASD, IB, VC, SH, MQ, TD, JD, JWT, ZKHS, DAR, FB, and MR collected samples and clinical data from patient and control cohorts. WA, KL, LCG, BAH, DMO, CHLS, CA, M. Bidlingmaier, and BGK performed biochemical analysis. YR, AF, PL, FB, and AKW performed immunostaining analysis. WA, KL, AJS, IB, M. Biehl, AKW, FB, and MR undertook data analysis. AJS, KL, and WA performed statistical analysis of results, with JJD providing statistical oversight. WA drafted the manuscript and all authors contributed to writing the manuscript and approved the version to be published.

## Supplementary Material

Supplemental data

ICMJE disclosure forms

## Figures and Tables

**Table 2:**
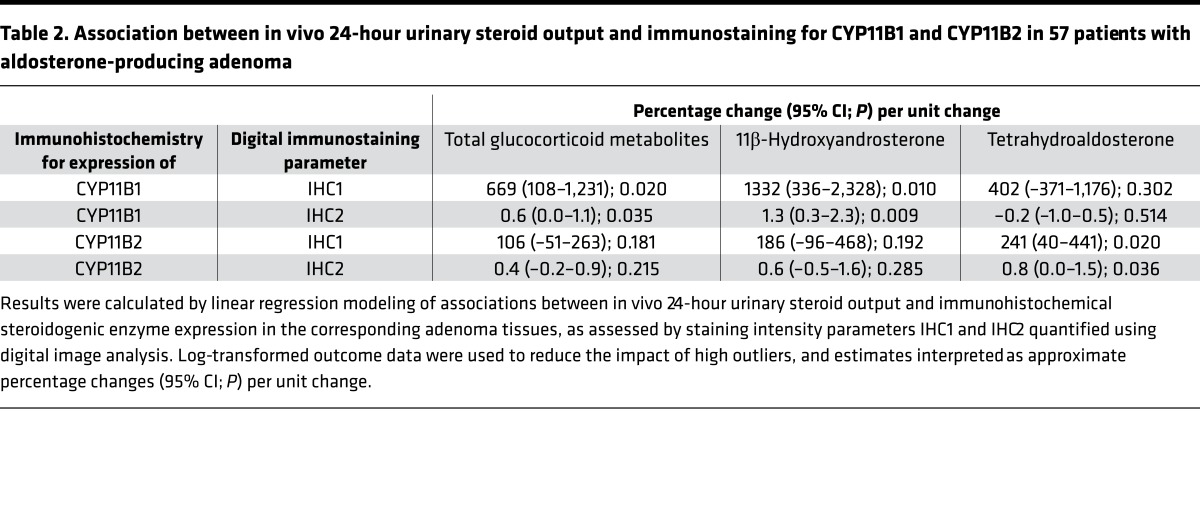
Association between in vivo 24-hour urinary steroid output and immunostaining for CYP11B1 and CYP11B2 in 57 patients with aldosterone-producing adenoma

**Table 1:**
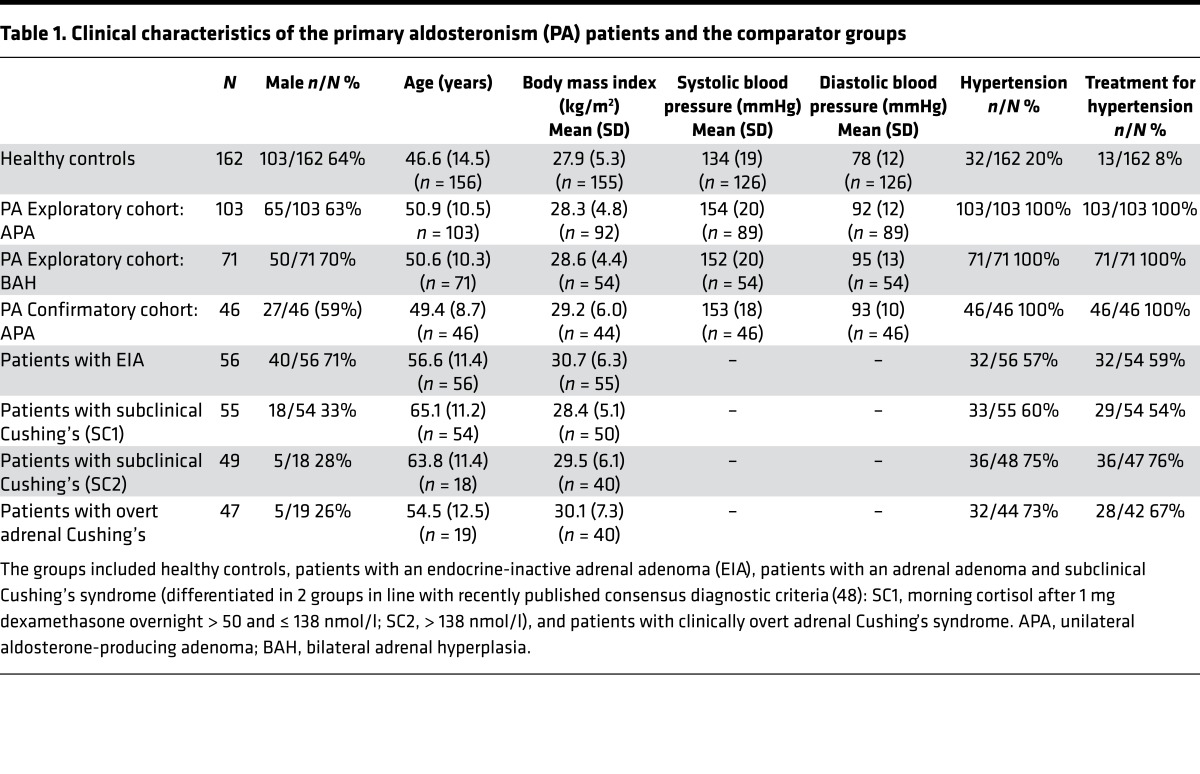
Clinical characteristics of the primary aldosteronism (PA) patients and the comparator groups
