# Supplementary material for: Steroid metabolome analysis reveals prevalent glucocorticoid excess in primary aldosteronism
Source: JCI Insight. 2017 Apr 20;2(8):e93136. doi: 10.1172/jci.insight.93136 (PMC5396526; doi:10.1172/jci.insight.93136)
Supplement: Supplemental data [file jciinsight-2-93136-s001.pdf]

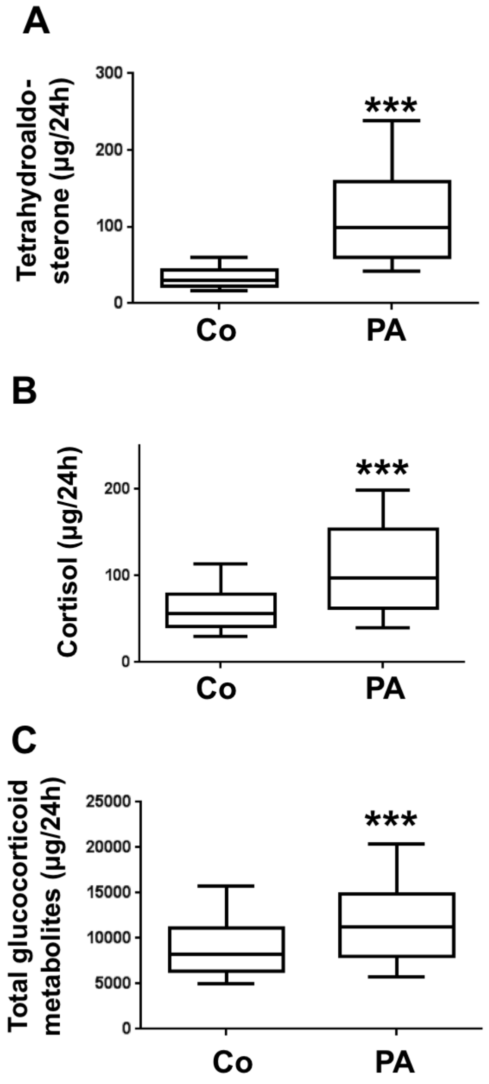

Figure S1: Comparison of 24-h urinary steroid metabolite excretion in 162 healthy controls and 174 patients with primary aldosteronism. Panel A, tetrahydroaldosterone; Panel B, cortisol; Panel C, total glucocorticoid metabolites. *** P<0.001 vs. controls. Comparisons between groups were made with linear regression models to adjust for age and sex in comparisons between the two groups.

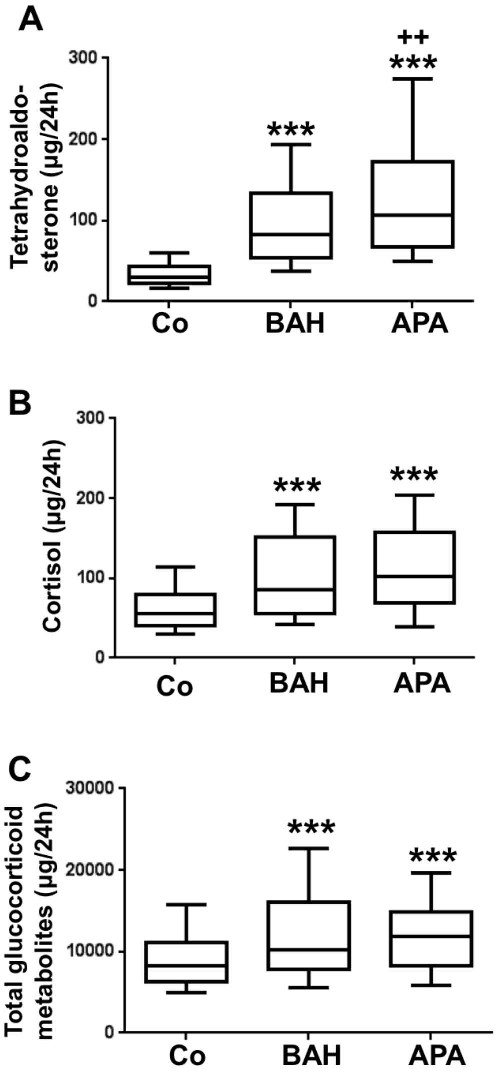

Figure S2: Comparison of 24-h urinary steroid metabolite excretion in healthy controls (n=162) and primary aldosteronism patients due to bilateral adrenal hyperplasia (BAH; n=71) or unilateral aldosterone-producing adrenal adenoma (APA; n=103). Panel A, tetrahydroaldosterone; Panel B, cortisol; Panel C, total glucocorticoid metabolites. *** P<0.001 vs. controls; ++ P<0.01BAH vs. APA. Comparisons between groups were made with linear regression models to adjust for age and sex in comparisons between the three groups.

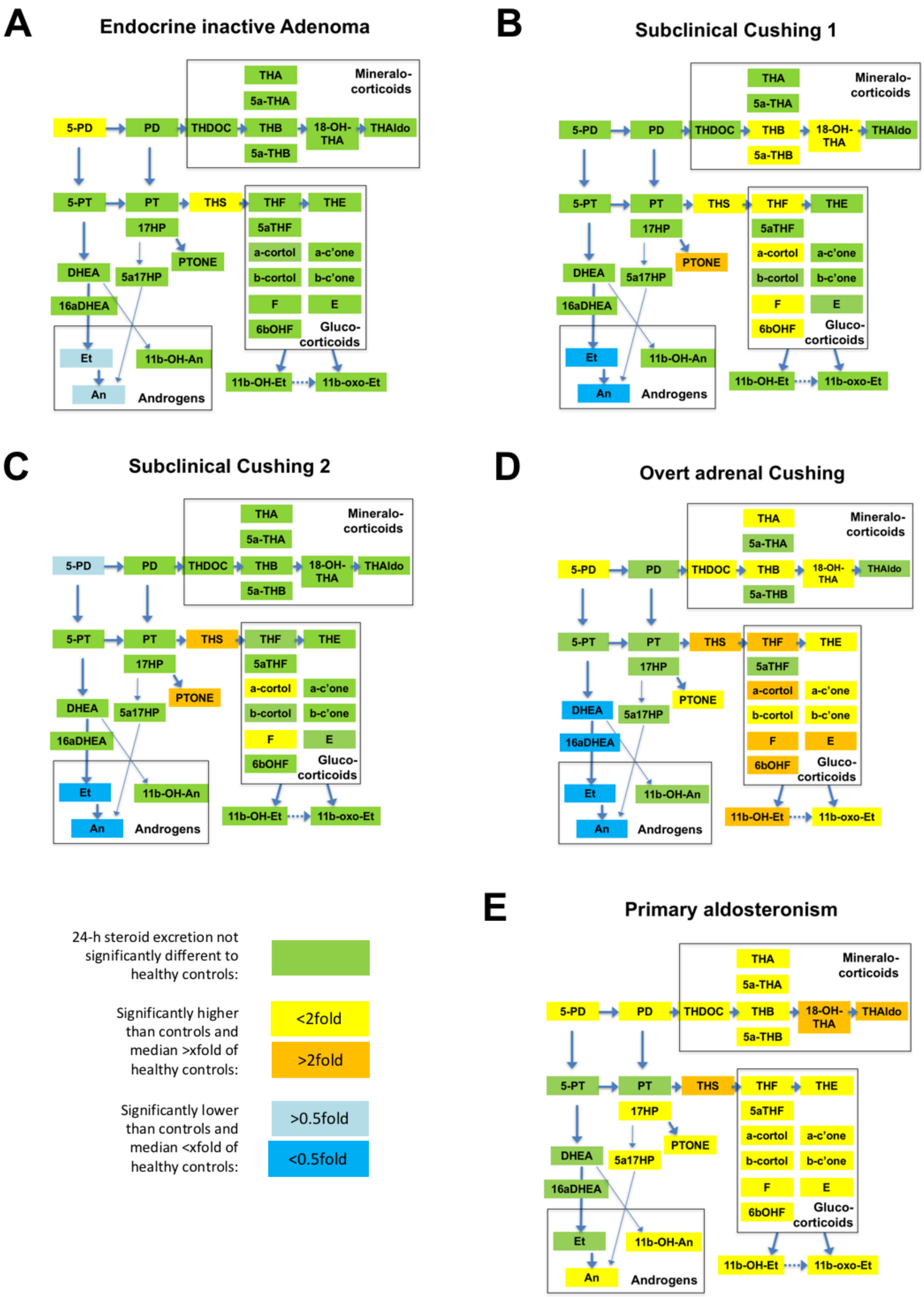
A
Endocrine inactive Adenoma
B
Subclinical Cushing 1
C
Subclinical Cushing 2
D
Overt adrenal Cushing
E
Primary aldosteronism
THA
5a-THA
Mineralo-corticoids
5-PD
PD
THDOC
THB
18-OH-THA
THAldo
5a-THB
5-PT
PT
THS
THF
THE
17HP
5aTHF
a-cortol
a-c'one
b-cortol
b-c'one
PTONE
DHEA
5a17HP
16aDHEA
F
E
6bOHF
Gluco-corticoids
Et
11b-OH-An
An
Androgens
11b-OH-Et
11b-oxo-Et
24-h steroid excretion not significantly different to healthy controls:
Significantly higher than controls and median >xfold of healthy controls:
<2fold
>2fold
Significantly lower than controls and median <xfold of healthy controls:
>0.5fold
<0.5fold

Figure S3: Schematic visualization of steroid metabolite pathway analysis. The figure depicts the metabolites mapped onto the adrenal steroidogenic pathways leading to mineralocorticoid, glucocorticoid and adrenal androgen precursor synthesis in patients with primary aldosteronism (n=174; Panel A), overt adrenal Cushing's syndrome (n=47; Panel B), subclinical Cushing's SC1 (n=55) and SC2 (n=49) (Panels C+D) and patients with endocrine inactive adrenal adenoma (n=56; Panel D).

Steroids in the pathways are represented by their major metabolites; for explanation of the abbreviations, related steroids of origin and detailed statistical analysis, please see Suppl. Table 1. Green indicates steroid concentrations similar to those in healthy controls (n=162); significantly increased steroids are indicated by yellow (median <2fold of the median of healthy controls) and orange (>2fold); significantly decreased steroid metabolites are indicated in light blue (median decreased to more than 0.5fold of the median of healthy controls) and dark blue (>0.5fold). Significant differences were determined by a linear regression model with the log transformed steroid metabolite as the outcome (adjusted for age and sex).

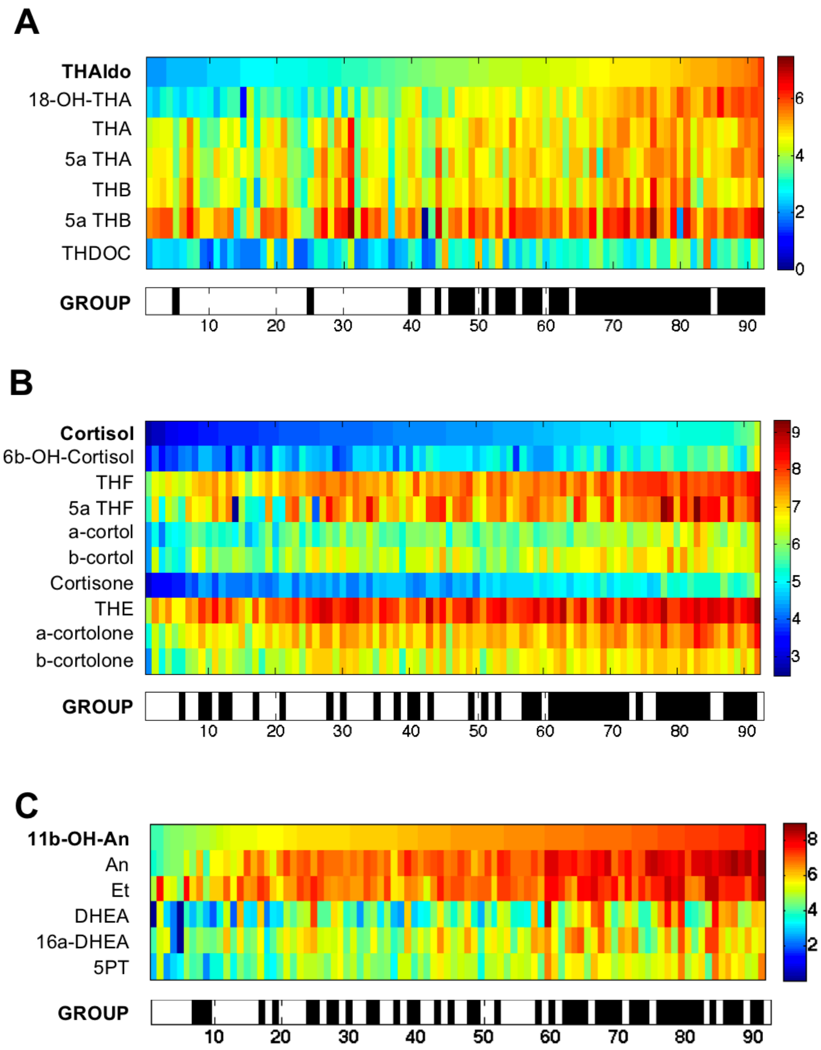

Figure S4: Heat map visualizations of steroid metabolome profiling in 46 primary aldosteronism patients comparing pre-operative (group: black) and post-operative (group: white) 24-h urinary steroid excretion. 24-h urines were collected one to two weeks after unilateral adrenalectomy for aldosterone-producing adrenal adenoma; steroid profiling was carried out by gas chromatography-mass spectrometry in selected-ion-monitoring mode.

Panel A, mineralocorticoid and mineralocorticoid precursor metabolites ordered according to increasing amounts of tetrahydroaldosterone excretion; Panel B, glucocorticoid metabolites in order of increasing amounts of cortisol excretion, and Panel C, selected androgen and androgen precursor metabolites ordered according to increasing androsterone excretion.

ppl. Table S1: Association between metabolic phenotype parameters (mean (standard deviation) or median [interquartile range]) in ients with primary aldosteronism and total glucocorticoid metabolite and tetrahydroaldosterone excretion, respectively, assessed ng linear regression models and adjustment for age and sex. Log transformed outcome data were used to reduce the impact of h liers, and estimates interpreted as approximate percentage changes (95% CI; p) per unit change in the metabolic risk parameter.).01 in bold. oGTT, oral glucose tolerance test (75 g glucose orally at 0 min); * mean (SD, standard deviation) or median [interquar ge (IQR)] dependent on whether data were normally distributed

	Primary aldosteronism patients (n=174)				Control subjects (n=162)			
**abolic risk ameters**	Total n (m/f)	mean (SD) or median (IQR*)	Percentage change in 24-h total glucocorticoid excretion per metabolic risk parameter unit	Percentage change in 24-h tetrahydroaldo-sterone excretion per metabolic risk parameter unit	Total n (m/f)	mean (SD) or median [IQR*]	Percentage change in 24-h total glucocorticoid excretion per metabolic risk parameter unit	F cha tetr ex me pa
y mass index ($/m^2$)	146 (91, 55)	28.4 (4.6)	2.5 (0.8, 4.2); **P=0.004**	1.1 (-1.2, 3.4); P=0.35	155 (102, 53)	27.0 [24.4, 30.8]	3.1 (1.9, 4.3); **P<0.001**	-1.
st circumference)	129 (81, 48)	99.0 (14.2)	1.3 (0.6, 2.0); **P<0.001**	0.3 (-0.6, 1.2); P=0.56	45 (28, 17)	108.0 [101.0, 115.5]	0.1 (-0.8, 0.9); P=0.90	0.6
. cholesterol /dL)	142 (88, 54)	53.0 (44.0, 65.0)	-0.9 (-1.4, -0.4); **P<0.001**	-0.4 (1.1, 0.2); P=0.21	76 (58, 18)	54.1 [46.4, 61.9]	0.1 (-0.7, 0.8); P=0.84	(
. cholesterol /dL)	141 (88, 54)	122.3 (34.5)	0.2 (0.0, 0.5); P=0.05	0.1 (-0.2, 0.4); P=0.38				
l cholesterol /dL)	142 (89, 53)	197.4 (38.9)	0.1 (-0.1, 0.3); P=0.41	0.01 (-0.2, 0.3); P=0.69	117 (82, 35)	197.2 (42.5)	0.1 (-0.1, 0.3); P=0.45	-0.
lycerides /dL)	143 (89, 54)	103.0 (76.0, 141.0)	0.1 (0.00, 0.2); P=0.08	0.01 (-0.1, 0.2); P=0.45	55 (38, 17)	115.1 [79.7, 159.4]	0.01 (-0.1, 0.1); P=0.88	0.1
ng glucose 0 min /dL)	112 (69, 43)	89.5 (82.5, 97.0)	0.0 (-0.9, 0.9); P=1.00	-0.3 (-1.3, 0.8); P=0.61	125 (88, 37)	88.3 (10.8)	0.1 (-0.6, 0.9); P=0.76	0.4
'T glucose 120 min /dL)	112 (69, 43)	123.5 (101.5, 162.0)	0.2 (-0.2, 0.2); P=0.86	0.01 (-0.2, 0.4); P=0.55	46 (28, 18)	124.3 [88.3, 158.5]	0.1 (-0.3, 0.4); P=0.76	0.1

ng insulin 0 min /mL)	107 (67, 40)	16.3 (9.9, 25)	0.8 (0.4, 1.3); **P=0.001**	0.1 (-0.5, 0.7); P=0.82	118 (81, 37)	6.4 [3.7, 11.1]	1.8 (0.8, 2.8); **P=0.001**	0.1
T insulin 120 min J/mL)	107 (67, 40)	77.3 (45.0, 152.0)	0.2 (0.1, 0.3); **P=0.003**	0.1 (-0.1, 0.2); P=0.32	45 (27, 18)	80.3 [45.5, 113.0]	0.3 (0.1, 0.5); **P=0.005**	-0.
neostasis Model essment of Insulin istance (HOMA-IR)	108 (67, 41)	3.8 (2.2, 5.9)	3.4 (1.3, 5.4); **P=0.001**	0.0 (-2.5, 2.6); P=0.98	116 (80, 36)	1.1 [0.6, 1.7]	19.6 (9.1, 30.1) **P<0.001**	3.1
$_{1c}$ (%)	98 (58, 40)	5.5 (0.5)	18.1 (1.8, 38.0); P=0.07	6.7 (-15.8, 29.2); P=0.56	41 (25, 16)	5.5 [5.2, 5.8]	4.6 (-20.4, 29.6) P=0.71	-17.
n systolic blood sure (mmHg)	143 (89, 54)	153 (20)	0.4 (-0.0, 0.8); P=0.07	0.6 (0.1, 1.1); P=0.02	126 (89, 37)	130 [119, 147]	-0.0 (-0.4, 0.4) P=0.99	0.
n diastolic blood sure (mmHg)	143 (89, 54)	93 (12)	0.9 (0.3, 1.5); **P=0.007**	0.3 (-0.5, 1.2); P=0.48	126 (89, 37)	79 [70, 87]	-0.3 (-1.0, 0.4) P=0.39	0.

pl. Table S2: Steroid metabolites—median [interquartile range] (µg/24h); p-values—measured by gas chromatography-mass spectrometry selec -monitoring analysis in in 24-h urine collected by primary aldosteronism patients, healthy controls and comparator cohorts with adrenal patholo p-values show the significance level when comparing each group with healthy controls using a linear regression model with the log transfor oid metabolite as the outcome (adjusted for age and sex). P<0.01 in bold.

**Abbre-viation**	**Common name**	**Chemical name**	**Metabolite of**	**Healthy controls (n=162)**	**Endocrine inactive adenoma (n=56)**	**Primary aldosteronism patients (n=174)**	**Subclinical Cushing's 1 (n=55)**	**Subclinical Cushing's 2 (n=49)**	**Clini adren sy (**
An	Androsterone	5α-androstan-3α-ol-17-one	Androstene-dione, testosterone, 5α-dihydro-testosterone	1431 [825, 2556]	799 [525, 1445]; **0.003**	1788 [964, 3169]; 0.015	351 [134, 656]; **<0.001**	238 [83, 534]; **<0.001**	
Etio	Etiocholanolone	5β-androstan-3α-ol-17-one	Androstene-dione, testosterone	1272 [836, 2147]	856 [590, 1297]; **0.008**	1466.0 [833, 2495]; 0.131	415 [246, 706]; **<0.001**	417 [160, 916]; **<0.001**	
11β-OH-An	11β-hydroxy-androsterone	5α-androstan-3α,11β -diol-17-one	11β-hydroxy-androstene-dione	469.3 [329.6, 760.0]	416.0 [295.3, 720.3]; 0.329	740.5 [391.5, 1140.3]; **<0.001**	396.0 [270.5, 686.5]; 0.712	278.0 [166.0, 819.0]; 0.824	[2
DHEA	Dehydroepi-androsterone	5-androsten-3β-ol-17-one	DHEA, DHEAS	256 [65, 1094]	109 [46, 227]; 0.127	213 [69, 993]; 0.186	49 [22, 105]; 0.478	26 [17, 72]; 0.376	
16α-OH-DHEA	16α-hydroxy-DHEA	5-androstene-3β,16α-diol-17-one	DHEA, DHEAS	403 [178, 814]	248 [67, 526]; 0.290	344 [168, 739]; 0.618	87 [45, 278]; 0.322	98 [38, 191]; 0.474	
5-PT	Pregnenetriol	5-pregnene-3β,17-20α-triol	17-hydroxy-pregnenolone	191 [119, 386]	139 [81, 234]; 0.298	209 [129, 382]; 0.256	74 [49, 170]; 0.514	70 [44, 115]; 0.340	

5-PD	Pregnenediol*	5-pregnene-3β,20α-diol and 5,17,(20)-pregnadien-3β-ol	Pregnenolone	320 [167, 552]	346 [197, 624]; **0.001**	408 [268, 639]; **<0.001**	240 [96, 363]; **0.002**	144 [61, 252]; 0.081	
THDOC	Tetrahydro-11-deoxy-corticosterone	5β-pregnane-3α,21-diol-20-one	11-deoxy-corticosterone	14.0 [9.9, 24.3]	14.0 [9.0, 20.3]; 0.076	24.0 [14.0, 36.0]; **<0.001**	14.0 [8.0, 24.0]; 0.031	12.0 [8.0, 19.0]; 0.279	
THA	Tetrahydro-11-dehydro-corticosterone	5β-pregnane-3α,21-diol-11,20-dione	Cortico-sterone, 11-dehydro-corticosterone	93.6 [64.5, 142.8]	97.0 [72.0, 127.3]; 0.335	136.5 [93.0, 200.0]; **<0.001**	101.0 [61.5, 141.5]; 0.020	58.0 [36.0, 104.0]; 0.191	[
5α-THA	5α-tetrahydro-11-dehydro-corticosterone	5α-pregnane-3α,21-diol-11,20-dione	Cortico-sterone, 11-dehydro-corticosterone	90.0 [64.3, 125.0]	90.0 [54.3, 139.3]; 0.487	132.0 [74.3, 200.3]; **0.001**	81.0 [53.0, 118.5]; 0.667	62.0 [44.0, 77.0]; 0.497	[
THB	Tetrahydro-corticosterone	5β-pregnane-3α,11β,21-triol-20-one	Cortico-sterone	102.2 [69.0, 151.8]	130.5 [75.3, 175.3]; 0.103	138.5 [82.3, 200.5]; **0.001**	126.0 [78.0, 175.5]; **0.001**	105.0 [61.0, 157.0]; 0.980	[
5α-THB	5α-tetrahydro-corticosterone	5α-pregnane-3α,11β,21-triol-20-one	Cortico-sterone	235.0 [153.6, 371.9]	264.0 [142.8, 509.8]; 0.348	329.5 [196.5, 476.5]; **0.002**	287.0 [121.5, 421.5]; **0.008**	144.0 [64.0, 266.0]; 0.937	[
18-OH-THA	18-hydroxy-tetrahydro-11-dehydrocorti-costerone	5β-pregnane-3α,18,21-triol-11,20-dione	18-hydroxy-corticosterone	59.0 [38.0, 76.0]	59.5 [45.0, 80.8]; 0.414	136.5 [83.3, 235.5]; **<0.001**	59.0 [40.0, 94.0]; **0.007**	58.0 [37.5, 75.3]; 0.329	[
THAldo	3α,5β-tetrahydro-aldosterone	5β-pregnane-3α,11β,21-triol-20-one-18-al	Aldosterone	30.0 [22.3, 44.0]	31.5 [23.0, 45.0]; 0.552	98.5 [60.0, 156.8]; **<0.001**	27.5 [21.0, 41.8]; 0.685	29.0 [17.8, 40.0]; 0.914	

PD	Pregnanediol	5β-pregnane-3α,20α-diol	Progesterone	182.5<br>[108.3, 267.5]	150.5<br>[107.5, 244.8];<br>0.379	245.0<br>[131.0, 408.3];<br>**<0.001**	131.0<br>[89.0, 213.0];<br>0.928	132.0<br>[72.0, 201.0];<br>0.516	[1
3α-5α-17HP	5α-17-hydroxy-pregnanolone	5α-pregnane-3α,17α-diol-20-one	17-hydroxy-progesterone	14.2<br>[8.0, 25.8]	15.0<br>[7.0, 30.0];<br>0.152	22.0<br>[10.0, 35.8];<br>**<0.001**	7.0<br>[3.0, 14.5];<br>0.522	4.1<br>[3.0, 12.0];<br>0.472	
17HP	17-hydroxy-pregnanolone	5β-pregnane-3α,17α-diol-20-one	17-hydroxy-progesterone	121.0<br>[67.3, 229.8]	157.0<br>[84.3, 226.3];<br>0.017	195.5<br>[107.3, 261.0];<br>**<0.001**	63.0<br>[38.5, 127.0];<br>0.390	90.0<br>[51.0, 160.0];<br>0.161	[
PT	Pregnanetriol	5β-pregnane-3α,17α,20α-triol	17-hydroxy-progesterone	535.0<br>[306.0, 781.0]	444.0<br>[258.3, 606.0];<br>0.225	635.0<br>[372.5, 862.8];<br>0.022	278.0<br>[172.0, 433.5];<br>0.153	258.0<br>[178.0, 488.0];<br>0.265	[1
PTONE	Pregnane-triolone	5β-pregnane-3α,17α,20α-triol-11-one	21-deoxycortisol	11.0<br>[7.0, 16.3]	14.5<br>[9.8, 28.3];<br>0.058	20.5<br>[13.0, 35.8];<br>**<0.001**	26.0<br>[12.5, 39.5];<br>**<0.001**	22.0<br>[9.0, 51.0];<br>**0.005**	
THS	Tetrahydro-11-deoxycortisol	5β-pregnane-3α,17α,21-triol-20-one	11-deoxycortisol	60.0<br>[41.3, 86.8]	104.5<br>[67.5, 147.5];<br>**<0.001**	132.5<br>[82.3, 212.3];<br>**<0.001**	119.0<br>[80.0, 207.0];<br>**<0.001**	139.0<br>[74.0, 221.0];<br>**<0.001**	[1
F	Cortisol	4-pregnene-11β,17,21-triol-3,20-dione	Cortisol	55.7<br>[41.0, 78.8]	65.0<br>[45.8, 97.8];<br>0.034	97.5<br>[63.8, 153.0];<br>**<0.001**	74.0<br>[50.5, 115.5];<br>**<0.001**	72.0<br>[50.0, 98.0];<br>**0.007**	[1
6β-OH-F	6β-hydroxy-cortisol	4-pregnene-6β,11β,17,21-tetrol-3,20-dione	Cortisol	111.5<br>[77.2, 157.3]	114.5<br>[83.0, 165.0];<br>0.355	146.0<br>[95.0, 227.8];<br>**<0.001**	159.0<br>[83.5, 231.0];<br>**<0.001**	143.0<br>[81.0, 236.0];<br>0.096	[1

THF	Tetrahydro-cortisol	5β-pregnane-3α,11β,17,21-tetrol-20-one	Cortisol	1435.0 [1032.0, 1958.8]	1527.5 [1011.0, 2087.8]; 0.993	1941.0 [1337.8, 2677.3]; **<0.001**	1751.0 [1335.0, 2296.0]; **0.009**	1747.0 [1362.0, 2157.0]; 0.532	[197
5α-THF	5α-tetrahydro-cortisol	5α-pregnane-3α,11β,17,21-tetrol-20-one	Cortisol	1278.3 [738.5, 2073.0]	1207.0 [598.5, 2123.3]; 0.590	1687.0 [1065.8, 2672.0]; **0.001**	1148.0 [591.0, 1986.0]; 0.368	672.0 [272.0, 1410.0]; 0.928	[47
α-cortol	α-cortol	5β-pregnan-3α,11β,17,20α,21-pentol	Cortisol	291.0 [203.6, 384.0]	336.5 [233.3, 507.0]; 0.298	363.5 [264.3, 513.8]; **<0.001**	365.0 [260.0, 497.0]; **0.001**	354.0 [255.0, 440.0]; **0.002**	[42
β-cortol	β-cortol	5β-pregnan-3α,11β,17,20β,21-pentol	Cortisol	450.5 [285.5, 620.0]	445.5 [309.8, 693.0]; 0.497	613.5 [431.0, 838.3]; **<0.001**	503.0 [342.0, 629.5]; 0.013	452.0 [281.0, 672.0]; 0.090	[47
11β-OH-Et	11β-hydroxy-etiocholanolone	5β-androstan-3α,11β-diol-17-one	Cortisol	262.9 [121.0, 408.0]	261.0 [141.3, 441.5]; 0.952	298.0 [120.5, 579.8]; 0.219	306.0 [118.0, 511.5]; 0.719	340.0 [104.0, 517.0]; 0.249	[27
E	Cortisone	4-pregnene-17α,21-diol-3,11,20-trione	Cortisone	86.2 [67.5, 122.8]	91.5 [68.8, 121.3]; 0.397	130.5 [94.0, 180.3]; **<0.001**	90.0 [71.5, 129.0]; 0.030	97.0 [68.0, 133.0]; 0.464	[1
THE	Tetrahydro-cortisone	5β-pregnene-3α,17,21-triol-11,20-dione	Cortisone	2841.0 [2177.8, 3984.8]	2992.0 [1852.5, 4090.5]; 0.481	3554.5 [2618.3, 5265.0]; **0.001**	2839.0 [1932.5, 4159.5]; 0.324	2362.0 [1587.0, 3190.0]; 0.103	[224
α-cortolone	α-cortolone	5β-pregnane-3α,17,20α,21-tetrol-11-one	Cortisone	1066.0 [821.8, 1432.5]	1176.5 [793.0, 1568.8]; 0.606	1342.5 [916.5, 1840.3]; **0.001**	1099.0 [885.0, 1487.0]; 0.547	898.0 [730.0, 1217.0]; 0.707	[88

β-cortolone	β-cortolone	5β-pregnane-3α,17,20β,21-tetrol-11-one	Cortisone	563.0 [419.7, 797.3]	655.5 [424.3, 827.5]; 0.866	812.5 [572.5, 1129.0]; **<0.001**	611.0 [411.5, 849.5]; 0.049	500.0 [303.0, 701.0]; 0.639	[38
11-oxo-Etio	11-oxo-etiocholanolone	5β-androstan-3α-ol-11,17-dione	Cortisol, cortisone	319.0 [193.3, 505.3]	333.0 [175.5, 516.5]; 0.568	400.0 [245.3, 687.0]; **0.006**	351.0 [162.0, 503.5]; 0.903	237.0 [113.0, 471.0]; 0.083	[2
24-h glucocorticoid metabolite excretion ılated as the sum of F, 6β-OH-F, THF, 5α-THF, ːtol, β-cortol, THE, α-cortolone, β-cortolone)			Cortisol	8262.0 [6379.8, 11043.5]	8269.0 [5784.0, 12067.8]; 0.730	11306.0 [8042.3, 14868.8]; **<0.001**	9233.0 [6508.0, 12218.5]; 0.037	8390.0 [6437.0, 10889.0]; 0.788	

Suppl. Table S3: Modelling of 24-h urinary steroid output (tetrahydroaldosterone, cortisol, total glucocorticoid metabolite excretion, all log transformed). Results in the upper part of the table are for comparison of participants with primary aldosteronism (PA) with healthy controls, adjusting for age, sex and body mass index. The lower part of the table shows results for comparison of all four adrenal disease states, primary aldosteronism (PA), endocrine inactive adrenal adenoma (EIA), subclinical (SC1 amnd SC2) adenoma, and overt adrenal Cushing's syndrome (Cu) with healthy controls adjusting for age and sex. Values presented are percentage differences (each group compared with healthy controls) with 95% confidence interval (95% CI). P<0.01 in bold. Total glucocorticoids were calculated as the sum of cortisol, 6β-OH-cortisol, THF, 5α-THF, α-cortol, β-cortol, THE, α-cortolone, and β-cortolone (=steroids 21-26 and 28-31 in Suppl. Table S1).

	Percentage differences (95% CI); P-value	
24-h tetrahydroaldosterone excretion	(N=301)	
	PA	110.7 (97.8, 123.5); **<0.001**
24-h cortisol excretion	(N=301)	
	PA	45.8 (33.3, 58.2); **<0.001**
24-h total glucocorticoid* metabolite excretion	(N=301)	
	PA	22.0 (12.3, 31.7); **<0.001**
24-h tetrahydroaldosterone excretion	(N=476)	
	EIA	6.3 (-14.4, 26.9); 0.552
	PA	119.6 (105.3, 134.0); **<0.001**
	SC1	-4.7 (-27.6, 18.2); 0.685
	SC2	1.8 (-31.7, 35.4); 0.914
	Cu	-12.3 (-44.2, 19.7); 0.451
24-h cortisol excretion	(N=477)	
	EIA	19.6 (1.5, 37.7); 0.034
	PA	49.5 (36.9, 62.0); **<0.001**
	SC1	40.8 (20.9, 60.7); **<0.001**
	SC2	40.3 (10.9, 69.7); **0.007**
	Cu	161.0 (133.0, 189.0); **<0.001**
24-h total glucocorticoid metabolite excretion	(N=477)	
	EIA	-2.7 (-17.9, 12.5); 0.730
	PA	25.0 (14.4, 35.6); **<0.001**
	SC1	17.8 (1.1, 34.6); 0.037
	SC2	3.4 (-21.4, 28.1); 0.788
	Cu	81.4 (57.8, 104.9); **<0.001**

Suppl. Table S4: Steroid metabolites (median [interquartile range]; µg/24h) measured in 24-h urine by gas chromatography-mass spectrometry selected-ion-monitoring analysis in 162 healthy controls and 46 primary aldosteronism at time of diagnosis and three to six months after unilateral adrenalectomy for aldosterone-producing adrenocortical adenoma. Displayed with median [interquartile ranges] are p-values from models comparing preoperative and post-operative measures with control measures adjusted for age and sex. P-values for comparing pre-operative and post-operative measures are calculated using Wilcoxon signed-rank tests. P<0.01 in bold.

No	Abbre-viation	Healthy controls (n=162)	Primary aldosteronism pre-operative (n=46)	Primary aldosteronism post-operative (n=46)	p-value comparing pre-operative and post-operative
1	An	1431<br>[823, 2572]	1333<br>[99, 3057]<br>0.02	923<br>[442, 1360]<br>**0.004**	**<0.001**
2	Etio	1272<br>[835, 2154]	1099<br>[755, 1950]<br>0.46	990<br>[530, 1384]<br>**0.008**	**0.001**
3	11β-OH-An	469<br>[327.8, 764]	807<br>[393, 1186]<br>**<0.001**	404<br>[231, 698]<br>0.27	**<0.001**
4	DHEA	256<br>[64, 1116]	147<br>[50, 454]<br>0.74	50<br>[22, 130]<br>**<0.001**	**<0.001**
5	16α-OH-DHEA	403<br>[178, 823]	292<br>[107, 504]<br>0.93	161<br>[77, 261]<br>**<0.001**	**<0.001**
6	5-PT	191<br>[119, 387]	186<br>[127, 256]<br>0.73	126<br>[62, 179]<br>**0.002**	**<0.001**
7	5-PD	320<br>[167, 552]	339.5<br>[226, 526]<br>**<0.001**	302<br>[141, 515]<br>0.39	0.10
8	THDOC	14<br>[10, 24]	25<br>[18, 40]<br>**<0.001**	12<br>[6.2, 22]<br>0.84	**<0.001**
9	THA	94<br>[64, 143]	137<br>[94, 176]<br>**<0.001**	83<br>[43, 104]<br>**<0.001**	**<0.001**

10	5α-THA	90<br>[64, 125]	152<br>[92, 211]<br>**<0.001**	95<br>[52, 135]<br>0.99	**<0.001**
11	THB	102<br>[69, 152]	128<br>[94, 187]<br>**0.003**	89<br>[58, 132]<br>0.12	**<0.001**
12	5α-THB	235<br>[153, 376]	336<br>[208, 486]<br>**0.002**	232<br>[116, 365]<br>0.52	**<0.001**
13	18-OH-THA	59<br>[38, 76]	126<br>[97, 250]<br>**<0.001**	27<br>[18, 53]<br>**<0.001**	**<0.001**
14	THAldo	30<br>[22, 44]	101<br>[62, 150]<br>**<0.001**	19<br>[13, 31]<br>**0.001**	**<0.001**
15	PD	183<br>[108, 268]	216<br>[129, 393]<br>**0.002**	145<br>[78, 246]<br>0.70	0.04
16	3α-5α-17HP	14<br>[8, 26]	22<br>[9, 33]<br>**<0.001**	16<br>[4.2, 27]<br>0.18	**0.01**
17	17HP	121<br>[67, 230]	150<br>[90, 265]<br>**<0.001**	120<br>[74, 245]<br>0.09	**0.01**
18	PT	535<br>[306, 781]	623<br>[383, 829]<br>**0.001**	481<br>[327, 681]<br>0.48	**0.01**
19	PTONE	11<br>[7, 16]	22<br>[15, 43]<br>**<0.001**	19<br>[12, 42]<br>**<0.001**	0.80
20	THS	60<br>[41, 87]	156<br>[106, 236]<br>**<0.001**	76<br>[54, 113]<br>0.11	**<0.001**
21	F	56<br>[41, 79]	109<br>[69, 156]<br>**<0.001**	58<br>[45, 79]<br>0.32	**<0.001**
22	6β-OH-F	112<br>[77, 159]	145<br>[98, 233]<br>**<0.001**	101<br>[64, 135]<br>0.66	**0.01**
23	THF	1435<br>[1031, 1962]	2136<br>[1424, 2820]<br>**<0.001**	1569<br>[1054, 1927]<br>0.99	**<0.001**

24	5α-THF	1278 [735, 2076]	1788 [1026, 2694] **<0.001**	1250 [574, 2110] 0.66	**<0.001**
25	α-cortol	291 [204, 384]	407 [325, 522] **<0.001**	297 [205, 428] 0.38	**<0.001**
26	β-cortol	451 [285, 621]	671 [523, 910] **<0.001**	525 [334, 652] **0.01**	**0.001**
27	11β-OH-Et	263 [120, 409]	282 [96, 580] 0.26	257 [108, 406] 0.67	0.07
28	E	86 [67, 123]	136 [105, 185] **<0.001**	75 [60, 105] 0.23	**<0.001**
29	THE	2841 [2174, 3991]	3628 [2836, 5337] **0.001**	2916 [1671, 4011] 0.46	**<0.001**
30	α-cortolone	1066 [821, 1436]	1415 [1103, 1838] **<0.001**	1057 [745, 1566] 0.53	**<0.001**
31	β-cortolone	563 [418, 798]	869 [700, 1126] **<0.001**	620 [449, 990] 0.11	**0.001**
32	11-oxo-Etio	319 [193, 510]	419 [215, 706] **0.008**	305 [154, 556] 0.72	**0.002**
Total 24-h glucocorticoid metabolite excretion*		8262 [6355, 11064]	12518 [8944, 15516] **<0.001**	9184 [5550, 11175] 0.91	<0.001

* Total glucocorticoids were calculated as the sum of cortisol, 6β-OH-cortisol, THF, 5α-THF, α-cortol, β-cortol, THE, α-cortolone, and β-cortolone (=steroids 21-26 and 28-31 i
